# Perceptions and attitudes of South African physiotherapists towards National Health Insurance

**DOI:** 10.4102/hsag.v30i0.3017

**Published:** 2025-07-31

**Authors:** Sholena Narain, Desmond Mathye

**Affiliations:** 1 Department of Physiotherapy, School of Health Care Sciences, Sefako Makgatho Health Sciences University, Pretoria, South Africa

**Keywords:** National Health Insurance, NHI bill, NHI act, physiotherapy, universal health coverage, rehabilitation, healthcare policy

## Abstract

**Background:**

The National Health Insurance (NHI) Act 20 of 2023 aims for universal health coverage. However, rehabilitation professions, especially physiotherapy, had limited involvement during key phases of NHI policy development, including the Green and White Papers, pilot projects, and the NHI Bill. President Cyril Ramaphosa enacted the NHI Bill in May 2024.

**Aim:**

To assess South African physiotherapists’ perceptions and attitudes towards NHI, focusing on their perception of its objectives and implications for their profession.

**Setting:**

An online survey was conducted among 146 South African physiotherapists.

**Methods:**

A quantitative, non-experimental online survey was used.

**Results:**

The data analysis revealed significant demographic influences on perceptions regarding NHI. Gender, age and professional experience played a role in shaping responses. Male physiotherapists were more likely than their female counterparts to perceive NHI as a means of addressing past healthcare disparities and increasing universal coverage. Professional experience and qualifications also played a crucial role, with distinct perspectives based on respondents’ qualifications. Age influenced opinions on the impact of NHI on physiotherapists in private practice, with younger physiotherapists perceiving more negative impacts compared to older colleagues.

**Conclusion:**

Physiotherapists acknowledge NHI’s potential to address healthcare disparities, but express concerns about its implementation and impact. They advocate for more inclusive policymaking, better communication, and improved strategies to ensure NHI meets diverse healthcare needs nationwide.

**Contribution:**

Developing demographic-sensitive strategies and addressing resource allocation and infrastructure challenges are crucial to implementing NHI effectively.

## Introduction

On 15 May 2024, the President of the Republic of South Africa (RSA), Cyril Ramaphosa, signed the National Health Insurance (NHI) Bill (RSA 2019) into law in Pretoria, marking a significant step towards achieving universal health coverage (UHC) in South Africa and reforming the country’s healthcare system (RSA 2024). This transformative initiative led to the NHI Bill (RSA 2019) becoming the *NHI Act 20 of 2023* (RSA 2023).

The *NHI Act of 2023* aims to ensure that all citizens in South Africa have equal access to comprehensive healthcare services, regardless of their economic status (RSA 2023). This Act serves as a transformative mechanism to achieve UHC, addressing historical disparities in healthcare (RSA 2023). It marks a significant step towards creating a more inclusive healthcare system in the country (RSA 2023).

The NHI Fund, established under the *NHI Act of 2023*, was conceived to improve healthcare service delivery across South Africa. This fund is pivotal in strategically purchasing healthcare services, focusing on equitable resource distribution and enhancing overall service efficiency (RSA 2023). The detailed framework established by the Act outlines the Fund’s powers, functions and governance structures, ensuring that these objectives align with the broader goals of efficiency and equity in healthcare provisioning. Moreover, the Act embeds rigorous provisions to prevent unethical practices within the healthcare system, highlighting a commitment to uphold national and international human rights standards. This alignment is further exemplified by the Act’s reinforcement of South Africa’s commitment to improving the quality of life for all its citizens, reflecting the country’s constitutional obligations, particularly those outlined in section 27(1) of the Constitution of the RSA (1996). Additionally, it highlights South Africa’s adherence to its obligations under various international human rights conventions (RSA 2023, 2024).

This legislative framework not only seeks to optimise the healthcare landscape in terms of access and quality but also ensures that the administration of health services adheres strictly to ethical and legal standards, positioning NHI as a cornerstone in South Africa’s pursuit of UHC (Department of Health [DOH] 2015a; RSA 2023).

The NHI is designed as a publicly funded system that pools funds to provide access to quality health services for all citizens and legal residents of South Africa based on their health needs and not their ability to pay (RSA 2023). The purpose of NHI, as outlined in the Act, is to create a single, unified healthcare system that offers a comprehensive range of services, including preventive, promotive, curative, rehabilitative and palliative care. National Health Insurance aims to eliminate financial barriers to healthcare, thereby reducing the out-of-pocket expenses that currently burden many South Africans, particularly those in lower-income groups. This move is aligned with global commitments to UHC, as endorsed by the World Health Organization (WHO) and the United Nations’ Sustainable Development Goals (SDGs) (WHO 2018).

The journey towards NHI has been long and complex, marked by several important milestones. The initial step was the release of the Green Paper in August 2011, which served as the government’s first formal proposal for NHI (DOH 2011). This document outlined the vision for a restructured healthcare system and set the stage for public and stakeholder engagement (DOH 2011).

In April 2011, the government initiated NHI pilot projects nationwide in 10 districts (DOH 2015b; Matsoso & Fryatt 2013; Ogunbanjo 2013). These projects were designed to test the feasibility of different components of the NHI system, including the effectiveness of service delivery models, the capacity of healthcare infrastructure and the readiness of healthcare professionals and facilities to operate under the proposed system (DOH 2015b; Matsoso & Fryatt 2013; Ogunbanjo 2013). While these pilot projects provided valuable insights into the potential of NHI, they also uncovered significant challenges. Critical barriers to successful implementation were identified, including inadequate infrastructure, shortages of healthcare professionals, logistical constraints and the need for substantial financial investment (DOH 2015b; Matsoso & Fryatt 2013; Ogunbanjo 2013).

Following extensive consultations and feedback, a White Paper on NHI was released in December 2015 (DOH 2015a). The White Paper provided a more detailed policy framework, setting out NHI’s strategic objectives, principles and proposed structures (DOH 2015a). It also addressed some concerns raised during the Green Paper phase, such as the financing mechanisms and the role of private healthcare providers.

In 2018, the NHI Bill was introduced to the South African Parliament (RSA 2019). This Bill provided the legislative foundation required to implement NHI. It set out the legal framework for establishing the NHI Fund, which would be responsible for pooling funds and purchasing healthcare services for the population (RSA 2019). The Bill also details the governance structures and the roles and responsibilities of various stakeholders in the NHI system (RSA 2019). Despite various challenges, the South African government advanced with the legislative process, ultimately culminating in the signing of the *NHI Act* (RSA 2024).

During the development and piloting of the NHI, serious concerns were raised by various practitioners on social media platforms, where the current authors participated. These practitioners perceived the exclusion of physiotherapy and other rehabilitation professions from key policy formulation and implementation stages, despite opportunities for public participation through comments on documents published in the Government Gazette, including NHI-related policies (RSA 2025).

While this exclusion remains a matter of perception, evidence supports these concerns. Physiotherapists and other rehabilitation professionals play a critical role in healthcare, particularly in the recovery and management of chronic conditions, disabilities and post-surgical rehabilitation (WHO 2024). However, their involvement in the NHI’s development was minimal, with rehabilitation services neither meaningfully integrated into the pilot phases nor explicitly incorporated into the *NHI Act* (DOH 2011, 2015a; RSA 2019, 2023).

The perceived exclusion of rehabilitation services, including physiotherapy, from key aspects of the NHI is further reflected in the limited references to these services within the NHI Bill and the *NHI Act*. Rehabilitation was mentioned only twice, specifically in the definitions of ‘comprehensive healthcare services’ and ‘basic healthcare services’ (RSA 2019, 2023). Before these legislative developments, the Green Paper referenced rehabilitation four times and ‘rehabilitative’ three times, primarily concerning health benefits, the pooling of funds, universal coverage, the comprehensive healthcare package and primary healthcare services (DOH 2011). Rehabilitation was mentioned only once in the context of district hospital services (DOH 2011). The minimal inclusion of rehabilitation in these foundational documents contributes to the perception that physiotherapy and other rehabilitation professions were not adequately considered in the design and implementation of the NHI. This perceived oversight raises concerns about the comprehensiveness and inclusivity of the NHI framework, potentially affecting its ability to deliver genuinely holistic healthcare services.

Whether excluding physiotherapy from the development of NHI is real or perceived, all healthcare practitioners, including physiotherapists, will be expected to adhere to the NHI framework and operate within the system. Therefore, this study aimed to investigate the perceptions and attitudes of South African physiotherapists towards NHI.

The main research question for this study was: ‘What are the perceptions and attitudes of South African physiotherapists towards NHI?’ The primary aim was to evaluate how well physiotherapists perceived NHI and to examine their views on its possible effects on their profession and the healthcare system. By assessing the perceptions and attitudes of these professionals, the study aimed to provide insights into how NHI could affect physiotherapy practices and healthcare delivery across South Africa.

## Research methods and design

### Study design

A quantitative, non-experimental online-based survey (Singh & Sagar 2021) was used on South African-based physiotherapists.

### Setting

The study was conducted across all provinces of the RSA as it was based online.

### Study population and sampling strategy

The study sample consisted of 146 South African physiotherapists registered with the Health Professions Council of South Africa (HPCSA), comprising both male and female physiotherapists working in the private, public and academic sectors across all provinces in the country. A convenience sample of registered physiotherapists with over 2 years of clinical experience completed an online questionnaire (survey) (Regmi et al. 2016). To ensure that respondents met the criterion of having at least 2 years of professional practice, the survey initially required participants to specify both the year of their first registration with the HPCSA and the year of commencement of their clinical practice. Only those who indicated a minimum of two full years in practice were permitted to proceed to the main questionnaire. To maintain respondent anonymity, no personally identifiable information, such as names, contact details or registration numbers, was collected. The authors placed trust in each participant’s professional integrity to accurately report their years of experience, thereby upholding both respondent confidentiality and the study’s inclusion criterion.

The research project was advertised on the Facebook pages of the two physiotherapy associations in South Africa. The Raosoft sample size calculator^1^ was used to determine the sample size. This calculation considered a population base of approximately 5000 registered physiotherapists. This figure was derived from the HPCSA 2015/2016 annual report (HPCSA 2016), which listed 6890 registered physiotherapists. The 5000 figure reflects the estimated number of physiotherapists with more than 2 years of clinical experience, after accounting for approximately 500 newly qualified physiotherapists produced annually by eight universities and about 1000 with less than 2 years of experience (HPCSA 2016). The sample size parameters included an 8% margin of error, a 95% confidence level and an assumed response distribution of 50%.

### Data collection

Data were collected using self-developed online surveys (questionnaires) and demographic information sheets between 03 February 2017 and 25 February 2017. Developing and testing the questionnaire included several stages to enhance its reliability and validity. Questionnaires were first reduced and streamlined to eliminate redundancy based on feedback from a research supervisor. The questionnaires were piloted by four physiotherapists to assess their accessibility and clarity, resulting in further refinements to enhance their quality. The internal reliability of the measuring instrument was evaluated using Cronbach’s alpha (α) coefficient. Taber (2018) suggested that a Cronbach’s alpha coefficient above 0.70 signifies that a questionnaire is valid and reliable. In this study, the Cronbach’s alpha coefficient for all sections completed by the 146 respondents was 0.860, exceeding the recommended threshold of 0.70 (Taber 2018). Lastly, peer reviews from physiotherapy lecturers at two universities helped identify areas for refinement.

The questionnaire used a 5-point Likert scale (Kusmaryono, Wijayanti & Maharani 2022), namely: 1 (strongly disagree), 2 (disagree), 3 (unsure), 4 (agree) and 5 (strongly agree), to measure respondents’ perceptions and attitudes. The following nine questions were responded to: (1) *NHI is the Government’s way of addressing the healthcare inequalities of the past;* (2) *NHI will increase universal coverage in South Africa;* (3) *NHI will provide affordable and quality health services;* (4) *Doctors played a dominant role in NHI pilot studies;* (5) *NHI will have a negative impact on physiotherapists in private practice;* (6) *NHI policy document has omitted the integral role of physiotherapy and rehabilitation;* (7) *The omission of physiotherapists in NHI pilot studies will have negative consequences in the implementation of NHI;* (8) *NHI services will be based on evidence-based practice and* (9) *Poor implementation, unclear logistics, lack of human and other resources and the state of the country are some of the reasons that NHI will not succeed in South Africa.*

### Data analysis

All data were uploaded to SPSS 24 for analysis. Descriptive statistics (frequencies and percentages) were used to present data, while inferential statistics helped explore deeper relationships. Although data were initially collected using a 5-point Likert scale, results were simplified into three categories: agree, unsure and disagree, for a more precise presentation.

The *t*-test was used to examine associations between demographic variables (such as gender, age, qualifications and current employment) and questions asked, assessing the differences between means. Pearson’s Chi-squared test (χ^2^) is a statistical tool applied to categorical data sets to assess the likelihood that any observed difference between the sets occurred by chance (Sullivan & Artino 2013). This study maintained a 5% significance level, with a *p*-value less than 0.05 indicating statistically significant differences, leading to the rejection of the null hypothesis. The null hypothesis posited that there is no statistically significant association or difference between demographic variables (such as gender, age, qualifications and current employment) and the respondents’ perceptions or attitudes regarding the items measured in the questionnaire. Furthermore, when significant differences between two means were found, Cohen’s *d* statistic was calculated to quantify the effect size of these differences (Bowring et al. 2021). For instance, in this study, Cohen’s *d* was used to assess differences in physiotherapists’ perceptions of NHI implementation based on their sector of employment (private vs. public). If the mean attitude score of physiotherapists working in the private sector was significantly higher than their counterparts in the public sector, a Cohen’s *d* value of 0.65 would indicate a moderate, practically significant difference between the two groups, implying meaningful implications for targeted policy interventions. According to Brydges (2019), Cohen’s *d* values between 0.20 and 0.50 indicate small yet meaningful differences, suggesting subtle variations in the responses of physiotherapists. When Cohen’s *d* values fall within the range of 0.50–0.80, they imply moderate practical significance, reflecting more pronounced differences in opinions among respondents. Values exceeding 0.80 are interpreted as indicating strong practical significance, which corresponds to substantial disparities in the responses. In contrast, Cohen’s *d* values below 0.20 suggest trivial differences, indicating that they have a negligible practical impact on the outcomes of the current study.

### Ethical considerations

Ethical approval was obtained from the Sefako Makgatho Health Sciences University Research Ethics Committee (reference no: SMUREC/H/178/2016:PG). All responses to the online questionnaires were accompanied by informed consent, as participants provided consent electronically when submitting their answers. To maintain confidentiality, names and identifiable features were not collected during the data collection process and are absent from the results reported in this article.

## Results

### Demographic information

Most respondents (78.8%) were female physiotherapists, with male physiotherapists comprising 21.2% of the sample. This gender distribution is consistent with findings from other South African studies, which also highlight a gender imbalance among registered physiotherapists (Louw et al. 2021). The respondents’ ages ranged from 23 to 64 years, with a mean of 36 years (s.d. = 11.21).

This average closely aligns with the findings of Hassem et al. (2022), who reported a mean age of 36–37 years. This average age suggests a relatively mature workforce within the physiotherapy profession.

Table 1 provides a summary of the respondents’ qualifications and employment sectors. Most respondents (71.9%) held a Bachelor’s degree in Physiotherapy, while 15.8% had a Master’s degree in Physiotherapy, which harmonises with Cobbing et al.’s (2017) and Hassem et al.’s (2022) research. A smaller proportion reported holding advanced qualifications, including a Doctor of Philosophy (PhD) (6.2%), an Master of Business Administration (MBA) (3.4%) and an Master of Public Health (MPH) (2.1%), with only 0.7% possessing a diploma. In terms of employment sectors, the largest proportion of respondents were employed in the private sector (43.8%), followed by the public sector (30.8%). About 14.4% were employed at universities. A smaller proportion reported working in two sectors (5.5%) or more than two sectors (2.1%), while 3.4% were categorised as working in ‘Other’ sectors.

**TABLE 1:** Qualifications and employment status.

Qualifications	*n*	%	Employment sector	*n*	%
BSc Physio	105	71.9	Private sector	64	43.8
MSc Physio	23	15.8	Public sector	45	30.8
MPH	3	2.1	University	21	14.4
MBA	5	3.4	Employed in two sectors	8	5.5
PhD	9	6.2	Employed in more than two sectors	3	2.1
Diploma	1	0.7	Other	5	3.4

**Total**	**146**	**100.0**	**-**	**146**	**100.0**

MPH, Master of Public Health; PhD, Doctor of Philosophy; MBA, Master of Business Administration.

Table 2 provides an overview of the respondents’ years of experience, year of qualification and province of employment. Respondents reported work experience ranging from 3 to 41 years, with an average of 13 years (s.d. = 1.5). The largest group (29.5%) had 5–10 years of experience, followed by those with 11–15 years (23.3%). A smaller proportion had less than 5 years of experience (13.0%), while 11.0% had over 25 years of experience. The qualification years ranged from 1974 to 2014, with most respondents (50.7%) qualifying between 2001 and 2010. Thirteen per cent qualified between 1995 and 2000, and 17.0% obtained their qualifications before 1995.

**TABLE 2:** Experience, qualification year and province.

Experience in years	*n*	%	Qualification year	*n*	%	Province	*n*	%
< 5 years	19	13.0	1970–1994	25	17.0	KwaZulu-Natal	38	26.0
5–10 years	43	29.5	1995–2000	19	13.0	Gauteng	43	29.5
11–15 years	34	23.3	2001–2010	74	51.0	Limpopo	9	6.2
16–20 years	24	16.4	2011–2014	28	19.0	Mpumalanga	6	4.1
21–25 years	10	6.8	-	-	-	Western Cape	28	19.2
> 25 years	16	11.0	-	-	-	Northern Cape	5	3.4
-	-	-	-	-	-	Eastern Cape	4	2.7
-	-	-	-	-	-	Free State	11	7.5
-	-	-	-	-	-	North West	2	1.4

**Total**	**146**	**100.0**	**-**	**146**	**100.0**	**-**	**146**	**100.0**

Respondents were distributed across all nine provinces, with the highest representation from Gauteng (29.5%), followed by KwaZulu-Natal at 26% and the Western Cape at 19.2%. These three provinces collectively represent nearly three-quarters of the total sample, indicating a strong urban-centric respondent base, possibly because of higher concentrations of health professionals and institutional networks in these regions. In contrast, Limpopo (6.2%) and Mpumalanga (4.1%) contributed smaller proportions. The Northern Cape (3.4%), Free State (7.5%) and Eastern Cape (2.7%) also had limited representation. The North West province reported the lowest participation rate, comprising only 1.4% of respondents.

### Perception and attitude towards National Health Insurance

Table 3 summarises respondents’ responses to the eight questions about their perceptions and attitudes towards NHI. The results demonstrate a range of views, reflecting both support and scepticism regarding the proposed NHI’s impact and implementation.

**TABLE 3:** Responses to eight questions.

Questions	Agree		Disagree		Unsure	
	*n*	%	*n*	%	*n*	%
1: *NHI is the Government’s way of addressing the healthcare inequalities of the past*	99	67.8	27	18.5	20	13.7
2: *NHI will increase universal coverage in South Africa*	60	41.1	42	28.8	44	30.1
3: *NHI will provide affordable and quality health services*	45	30.8	54	37.0	47	32.2
4: *Doctors played a dominant role in NHI pilot studies*	70	47.9	6	4.1	70	47.9
5: *NHI will have a negative impact on physiotherapists in private practice*	66	45.2	31	21.3	49	33.6
6: *NHI policy (Bill) document has omitted the integral role of physiotherapy and rehabilitation*	83	56.9	4	2.7	59	40.4
7: *The omission of physiotherapists in NHI pilot studies will have negative consequences in the implementation of NHI*	124	84.9	2	1.4	20	13.7
8: *NHI services will be based on evidence-based practice*	50	34.2	22	15.0	74	50.7

NHI, National Health Insurance.

A majority of respondents (67.8%) agreed that the ‘*NHI represents a governmental initiative aimed at addressing historical healthcare inequalities*’. In contrast, 18.5% disagreed with this view, while 13.7% were unsure. In response to the statement ‘*NHI will increase universal coverage in South Africa*’, the opinions were more divided. The 41.1% of respondents agreed, whereas 28.8% disagreed, and 30.1% were unsure, suggesting that a significant proportion of respondents either remain sceptical or uncertain about achieving UHC.

Regarding the statement ‘*NHI will provide affordable and quality health services*’, only 30.8% of respondents agreed. Thirty-seven per cent disagreed with the statement, while 32.2% were unsure, indicating limited confidence among respondents in the NHI’s ability to deliver both affordability and quality in health services.

On the question of professional involvement, respondents were asked whether ‘*Doctors played a dominant role in NHI pilot studies*’. Here, responses were almost evenly split: 47.9% agreed, 4.1% disagreed and another 47.9% were unsure. This suggests that while nearly half of the respondents perceived medical doctors as having led the pilot phase, an equal proportion remained uncertain. The high rate of uncertainty may indicate limited access to detailed information regarding the structure and leadership of the NHI pilot studies.

When presented with the statement ‘*NHI will have a negative impact on physiotherapists in private practice*’, 45.2% agreed, 21.3% disagreed and 33.6% were unsure. These findings reflect concerns among respondents about the potential negative implications of NHI for private physiotherapy practices. However, the significant level of uncertainty may indicate a need for more transparent policy communication in this area.

About the policy content, 56.9% of respondents agreed with the statement ‘*The NHI policy (Bill) document has omitted the integral role of physiotherapy and rehabilitation*’. Only 2.7% disagreed, while 40.4% were unsure. These responses suggest a strong perception that the policy inadequately acknowledges the role of physiotherapy and rehabilitation in the healthcare system. A majority (84.9%) agreed with the statement ‘*The omission of physiotherapists in NHI pilot studies will have negative consequences in the implementation of NHI*’, with only 1.4% disagreeing and 13.7% unsure. These findings highlight a widely shared concern that excluding physiotherapists from the pilot phase could undermine the effective implementation of NHI. Regarding whether ‘*NHI services will be based on evidence-based practice*’, 34.2% of respondents agreed, while 15.0% disagreed and 50.7% were unsure. This substantial level of uncertainty suggests that many respondents lack confidence or sufficient information about the extent to which evidence-based principles will be embedded in the NHI’s service delivery framework.

### Question 9: Factors responsible for the failure of National Health Insurance

The summary of responses related to Question 9 is presented in Table 4. The majority (35.6%) attributed the failure to a combination of all five factors: inability to finance NHI, poor implementation, unclear logistics, lack of human resources and the overall state of the country. About 22.6% highlighted four factors (inability to finance, poor implementation, unclear logistics and lack of human resources) as responsible. In comparison, 13.0% selected a combination of four factors that excluded unclear logistics but included the state of the country. Smaller groups identified combinations of three factors: 5.5% of the respondents identified inability to finance, poor implementation and unclear logistics, while 7.5% identified inability to finance, poor implementation and lack of human resources. Only a few respondents attributed the failure to individual factors, such as poor implementation (2.7%) or the state of the country (2.7%). About 8.2% of respondents expressed uncertainty about the causes.

**TABLE 4:** Question 9 – Factors responsible for the failure of National Health Insurance.

Factors	Frequency	Percentage
1. Inability to finance NHI	2	1.4
2. Poor implementation	4	2.7
3. Unclear logistics	-	-
4. Lack of human resources	1	0.7
5. State of the country	4	2.7
1, 2, 3, 4, 5 = Inability to finance, poor implementation, unclear logistics, lack of human resources and state of the country	52	35.6
1, 2, 3, 4 = Inability to finance, poor implementation, unclear logistics and lack of human resources	33	22.6
1, 2, 3 = Inability to finance NHI, poor implementation and unclear logistics	8	5.5
1, 2, 4 = Inability to finance NHI, poor implementation and lack of human resources	11	7.5
1, 2, 4, 5 = Inability to finance NHI, poor implementation, lack of human resources and state of the country	19	13.0
Unsure	12	8.2

NHI, National Health Insurance.

### Statistical associations between demographic variables and responses

The association between respondents’ demographic variables and their responses to the questions was evaluated using Pearson’s Chi-square (χ^2^) test, with statistical significance determined at a 5% level (*p* < 0.05). The questions’ mean scores and standard deviations (s.d.) were also calculated to understand the respondents’ overall agreement or uncertainty. Table 5 presents the detailed results of these analyses. Significant associations were found between specific demographic variables and selected questions. For instance, gender was significantly associated with Questions 1 (*p* = 0.000), 2 (*p* = 0.001), 3 (*p* = 0.004) and 4 (*p* = 0.002). Age showed a significant association with Question 5 (*p* = 0.050), while current employment was significantly associated with Questions 6 (*p* = 0.014) and 7 (*p* = 0.000). Qualification was significantly associated with Questions 3 (*p* = 0.021) and 8 (*p* = 0.008). The mean scores for Questions 1, 4, 5, 6, 7 and 9 ranged from 3.5 to 6.32, indicating agreement among respondents. In contrast, mean scores for Questions 2, 3 and 8 were below 3.5, reflecting uncertainty.

**TABLE 5:** Pearson Chi-square test for association between responses to the eight questions.

Variable	Questions								
	Q 1	Q 2	Q 3	Q 4	Q 5	Q 6	Q 7	Q 8	Q 9
Gender	0.000	0.001	0.004	0.002	0.757	0.048	0.806	0.145	0.891
Age	0.451	0.531	0.389	0.146	0.050	0.910	0.954	0.906	0.368
Province	0.069	0.702	0.225	0.472	0.352	0.214	0.603	0.182	0.680
Year of qualification	0.076	0.512	0.768	0.665	0.451	0.084	0.836	0.670	0.525
Current employment	0.642	0.296	0.224	0.080	0.422	0.014	0.000	0.105	0.971
Qualification	0.142	0.502	0.021	0.126	0.805	0.098	0.624	0.008	0.999
Years of experience	0.364	0.189	0.164	0.341	0.354	0.094	0.538	0.493	0.901
Mean	3.620	3.200	2.940	3.570	3.350	3.790	4.250	3.240	6.320
s.d.	1.038	1.124	1.122	0.769	1.021	0.849	0.738	0.865	2.020

s.d., standard deviation.

### T-tests for significant associations

Further analysis of significant associations was conducted using *t*-tests to evaluate differences between group means for demographic variables. Cohen’s *d* was calculated to assess the effect size, with thresholds for practical significance as follows: small (0.20 ≤ |*d*| < 0.50), moderate (0.50 ≤ |*d*| < 0.80) and large (|*d*| ≥ 0.80). A summary of the findings is presented in Table 6.

**TABLE 6:** Statistically significant associations between responses to the eight questions.

Category by demographic variable	Sub-category	*N*	Mean	s.d.	Mean difference (above diagonal) and Cohen’s value (*p*-value for *t*-statistic)				
					1	2	3	4	5
Q1 by Gender	1-Male	31	4.13	1.056	-	0.637 (0.002)	-	-	-
	2-Female	115	3.49	0.994	0.616 (0.04)	-	-	-	-
Q2 by Gender	1-Male	31	3.74	1.264	-	0.623 (0.002)	-	-	-
	2-Female	115	3.05	1.042	0.557 (0.008)	-	-	-	-
Q3 by Gender	1-Male	31	3.52	1.235	-	0.678 (0.001)	-	-	-
	2-Female	115	2.78	1.041	0.614 (0.004)	-	-	-	-
Q4 by Gender	1-Male	31	4.00	0.816	-	0.744 (0.000)	-	-	-
	1-Female	115	3.45	0.716	0.689 (0.001)	-	-	-	-
Q5 by Age (years)	1-20–30	31	3.55	1.060	-	0.160 (0.443)	0.801 (0.012)	0.048 (0.904)	0.511 (0.489)
	2-31–40	89	3.38	1.028	0.158 (0.452)	-	0.629 (0.023)	0.117 (0.752)	0.370 (0.606)
	3-41–50	16	2.75	0.856	0.859 (0.008)	0.715 (0.015)	-	0.907 (0.048)	0.277 (0.716)
	4-51–60	8	3.50	0.756	0.058 (0.885)	0.151 (0.692)	0.950 (0.044)	-	0.577 (0.486)
	5-61–70	2	3.00	1.414	0.393 (0.680)	0.272 (0.768)	0.183 (0.845)	0.382 (0.705)	-
Q3 by Qualification	1-BSc Physio	105	3.03	1.069	-	0.497 (0.033)	0.917 (0.120)	0.781 (0.091)	0.131 (0.707)
	2-MSc Physio	23	2.48	1.275	0.443 (0.064)	-	1.247 (0.053)	0.228 (0.647)	0.337 (0.399)
	3-MPH	3	4.00	0.000	5.467 (0.000)	3.512 (0.000)	-	2.634 (0.011)	1.178 (0.108)
	4-MBA	5	2.20	0.837	0.781 (0.090)	0.299 (0.560)	3.515 (0.009)	-	0.699 (0.235)
	5-PhD	9	2.89	1.054	0.132 (0.712)	0.366 (0.364)	2.110 (0.013)	0.749 (0.209)	-

MPH, Master of Public Health; PhD, Doctor of Philosophy; MBA, Master of Business Administration; s.d., standard deviation.

#### Gender and responses

*Question 1:* Male respondents showed stronger agreement than female respondents that NHI addresses past healthcare inequalities (*t*-test *p* = 0.002; Cohen’s *d* = 0.637, moderate). *Question 2:* Male respondents were more likely to agree that NHI increases universal coverage (*t*-test *p* = 0.002; Cohen’s *d* = 0.623, moderate). *Question 3:* Male respondents believed more strongly than female respondents that NHI would provide affordable, quality health services (*t*-test *p* = 0.001; Cohen’s *d* = 0.678, moderate). *Question 4:* Male respondents agreed more strongly than female respondents that doctors played a dominant role in NHI pilot studies (*t*-test *p* = 0.000; Cohen’s *d* = 0.744, moderate).

#### Age and responses

**Question 5:** Respondents aged 20–30 years were more likely than those aged 41–50 years to perceive that NHI negatively impacts physiotherapists in private practice (*t*-test *p* = 0.012; Cohen’s *d* = 0.801, moderate).

#### Qualification and responses

**Question 3:** A small but significant difference was observed between respondents with Bachelor’s and Master’s degrees regarding the affordability and quality of NHI (*t*-test *p* = 0.033; Cohen’s *d* = 0.497, small). However, respondents with an MPH displayed a significantly stronger belief in NHI’s affordability and quality than those with an MBA (*t*-test *p* = 0.011; Cohen’s *d* = 2.634, large).

## Discussion

The research findings indicated a substantial presence of physiotherapists in South Africa’s urban provinces, particularly in Gauteng, KwaZulu-Natal and Western Cape. Most of the respondents were female, constituting 78.8% of the total sample, with varying work experience ranging from 3 to 41 years and a large proportion engaged in private practice. These findings are in line with other research in South Africa. Studies have previously emphasised a similar gender imbalance among registered physiotherapists (Louw et al. 2021). Analysis of qualifications revealed that 71.9% held a Bachelor’s degree, while 15.8% and 6.2% held Master’s and PhD degrees, respectively, which complements other studies (Cobbing et al. 2017; Hassem et al. 2022).

The perceptions of South African physiotherapists regarding NHI demonstrated a mix of optimism and concern about its potential to enhance healthcare equity, effectiveness and service delivery. Although 67.8% regarded the NHI as essential for addressing historical healthcare disparities, consistent with governmental pledges for equitable healthcare access (Shezi 2023), only 41.1% felt that NHI would effectively broaden healthcare access. These reservations are further supported by Mukwena and Manyisa (2022), who identified significant infrastructure and resource allocation gaps that could hinder UHC. The NHI’s strategic approach must include substantial improvements in infrastructure and resources to tackle these challenges. Upgrading healthcare facilities, ensuring a sufficient supply of medical provisions, and enhancing technological systems are essential steps to align NHI’s ambitious goals with the realities of healthcare delivery (Mukwena & Manyisa 2022).

In addition, concerns persist about NHI’s capacity to ensure quality and affordable healthcare services, with only 30.8% of respondents expressing confidence in this aspect. Similar apprehensions were reported by Muofhe, Makwakwa and Motloba (2023), where oral health professionals expressed reservations about NHI’s impact on service quality, particularly within the private sector. This sentiment highlights apprehension about NHI’s resource management capabilities. Enhancing the transparency of NHI operations and communicating its benefits more clearly is essential to address these concerns. Mukwena and Manyisa (2022) and Muofhe et al. (2023) reiterated that clear, consistent and transparent communication is crucial to align policy objectives with public expectations and foster greater NHI acceptance. Moreover, for the inclusivity and success of NHI, the implementation process must involve a broad spectrum of healthcare professionals, not limited to medical doctors.

Approximately half of the respondents viewed medical doctors as dominant in NHI pilot studies, indicating a potentially marginalising top-down approach that excludes other essential healthcare professionals. This observation aligns with Shezi’s (2023) intuitions on excluding other healthcare professional roles in NHI’s planning processes. Shezi (2023) advocates for regular consultations with professionals from various sectors, including physiotherapists, to enrich NHI policies with diverse insights and expertise, enhancing their applicability and efficacy. Furthermore, concerns were raised about the potential negative impact on physiotherapists in private practice, with 45.2% of the respondents expressing apprehension about adverse effects. Such expression highlights broader concerns within the healthcare community about NHI’s implications for private healthcare providers (Muofhe et al. 2023). The reported expression emphasises the need for inclusive policies that effectively integrate private practitioners into the state-run system. Furthermore, the limited role of physiotherapy in critical aspects of NHI policy formulation, as critically viewed by 56.9% of respondents, emphasises the need for more comprehensive healthcare planning encompassing all facets of healthcare provision (Govender & Mahomed 2020). An overwhelming 84.9% of respondents indicated that excluding physiotherapists from NHI implementation planning could have adverse effects on the programme, highlighting the essential role of these professionals in healthcare delivery. Additionally, 34.2% of respondents expressed uncertainty regarding adherence to evidence-based practices in NHI implementations, pointing to the necessity for transparent and robust clinical governance frameworks to ensure NHI operations’ reliability and scientific foundation.

The data analysis also revealed demographic influences on the perceptions of NHI. Gender, age and professional experience significantly impacted responses. Specifically, gender differences were evident in attitudes towards NHI’s role in addressing healthcare inequalities and its potential to enhance UHC. For instance, male physiotherapists were more likely than female physiotherapists to believe in NHI’s effectiveness in addressing past healthcare disparities and increasing UHC. Furthermore, professional experience and qualifications also played a crucial role, with different levels of agreement on NHI’s ability to provide affordable and quality healthcare based on respondents’ qualifications. For example, respondents with master’s or a doctoral degree demonstrated more favourable views towards the affordability and quality of healthcare under the NHI compared to those holding only bachelor’s degrees in physiotherapy. A small but statistically significant difference was observed between Bachelor’s and Master’s degree holders, with Master’s graduates expressing greater confidence in the NHI’s ability to provide affordable, quality care. Respondents with a MPH degree held a markedly stronger belief in the affordability and quality of NHI services than those with a MBA.

Age also influenced opinions on the impact of NHI on physiotherapists in private practice, with younger physiotherapists perceiving a more negative impact compared to older colleagues. The finding that younger physiotherapists perceive the NHI as having a more negative impact on private practice than their older counterparts suggests a generational divide in attitudes towards healthcare reform. Younger practitioners, who are still establishing their careers, may feel more vulnerable to changes that could impact their professional autonomy and financial stability. In contrast, older physiotherapists may feel more secure or adaptable because of their experience.

These demographics significantly affect their expectations and experiences within the healthcare system. To address these demographic variations, it is imperative to tailor approaches that cater to different demographic groups’ unique needs and concerns. This tailored approach will help mitigate healthcare access and outcome disparities, ensuring equitable and effective service provision under NHI.

Various interrelated factors, including financing, implementation logistics and resource availability, may present potential barriers to NHI implementation (Mukwena & Manyisa 2022). These factors emphasise the necessity for a comprehensive approach that involves all stakeholders, ensures adequate resource allocation, upholds high standards of healthcare delivery and fosters broad understanding and support among healthcare professionals.

### Strengths and limitations of the study

To the best of our knowledge, this study is the first investigation into South African physiotherapists’ perceptions and attitudes towards NHI. Using a convenience sample may limit the generalisation of findings to South African physiotherapists. An online survey could have excluded those lacking digital access or those less likely to participate, introducing selection bias, especially among older physiotherapists. Advertising participation on social media might have missed practitioners who were not active on these platforms. Additionally, given that the study was conducted in 2017, it is possible that physiotherapists’ views on the NHI may have changed with its transition from the NHI Bill to the *NHI Act* signed by President Cyril Ramaphosa.

### Implications and recommendations

The impact of demographic factors such as gender, age and professional experience on physiotherapists’ perceptions of NHI highlights the importance of demographic-sensitive strategies in implementing it to address the diverse needs of all South Africans. The findings also revealed that the limited involvement of physiotherapists in NHI policy formulation and pilot studies could undermine the programme’s success, suggesting the need for their greater inclusion, alongside other rehabilitation professionals, to ensure comprehensive healthcare delivery. Additionally, the study reaffirmed concerns regarding resource allocation, infrastructure and the practical challenges of implementing NHI, emphasising the necessity of addressing these issues to translate NHI’s theoretical benefits into tangible improvements in healthcare access and quality. Regular assessments should be established to promptly identify and address emerging challenges, ensuring that NHI remains adaptable and responsive to the needs of its stakeholders.

## Conclusion

Physiotherapists acknowledge the potential of NHI to address enduring healthcare inequities in South Africa; nevertheless, their endorsement is tempered by profound concerns regarding its structural design, implementation and inclusivity. The findings of this study reveal a profession that is cautiously optimistic yet acutely aware of the policy’s deficiencies, particularly concerning communication gaps, exclusion from pilot initiatives and the perceived marginalisation of rehabilitation services in official documents. Specific concerns articulated by respondents pertain to implementation strategies, including the exclusion of physiotherapists from NHI pilot studies, the lack of clarity concerning reimbursement models for private practitioners and the insufficient recognition of rehabilitation services in the NHI Bill. These issues have practical ramifications: excluding physiotherapists from initial policy trials limits their contribution to refining service models, vague funding mechanisms may jeopardise the viability of private practices and neglecting rehabilitation in policy language may imperil the resourcing of a critical component of comprehensive care. Such strategies not only undermine professional confidence but also risk diminishing access to diverse and high-quality physiotherapy services across the healthcare continuum. These findings underscore the critical and immediate necessity for a sophisticated and inclusive policy dialogue that fully acknowledges and integrates the rich array of professional perspectives within the extensive and diverse healthcare workforce. The varying degrees of confidence, concern and uncertainty regarding the NHI, influenced by factors such as educational background and career stage, illustrate the complexity of implementing reform in pluralistic health systems. For the NHI to be successful, it must transcend a one-size-fits-all approach and actively engage stakeholders across different demographic and professional strata. Absent this, the reform risks entrenching mistrust and alienating essential contributors to its success. Ultimately, physiotherapists advocate for more inclusive policymaking, transparent communication and targeted implementation strategies that affirm their role within the health system. These measures are essential not only for attaining professional buy-in but also for ensuring that NHI evolves into an authentically equitable and functionally integrated healthcare model.

## Footnotes

1 http://www.raosoft.com/samplesize.html.
